# Assessing the risk of autochthonous yellow fever transmission in Lazio, central Italy

**DOI:** 10.1371/journal.pntd.0006970

**Published:** 2019-01-10

**Authors:** Mattia Manica, Giorgio Guzzetta, Federico Filipponi, Angelo Solimini, Beniamino Caputo, Alessandra della Torre, Roberto Rosà, Stefano Merler

**Affiliations:** 1 Department of Biodiversity and Molecular Ecology, Research and Innovation Centre, Fondazione Edmund Mach, San Michele all’Adige, Trento, Italy; 2 Center for Information Technology, Fondazione Bruno Kessler, Trento, Italy; 3 Epilab-JRU, FEM-FBK Joint Research Unit, Trento, Italy; 4 Department of Public Health and Infectious Diseases, Laboratory affiliated to Istituto Pasteur Italia–Fondazione Cenci Bolognetti, Sapienza University of Rome, Rome, Italy; University of California, Davis, UNITED STATES

## Introduction

Yellow fever virus (YFV) causes a highly lethal mosquito-borne disease that has recently reemerged after 30 years of low incidence due to vaccination campaigns. Large epidemics occurred in Angola and Democratic Republic of the Congo (DRC) in 2016 through 2017 (overall about 1,000 confirmed cases and 140 deaths) and in Brazil in 2017 through 2018 (2,037 confirmed cases and 674 deaths) [1, 2]. The high international connectivity of Brazil raises concern about the potential spread of disease to other countries by infected travelers [3, 4]; this possibility was confirmed during spring 2018, with the notification of six infected travelers from five European countries, two of which had a fatal outcome [5]. Recent laboratory experiments suggest that European populations of *Aedes albopictus* may be competent for transmission of YFV [6], and therefore large areas highly infested by this species in Mediterranean countries are potentially exposed to the risk of outbreaks [7].

Here, we provide a quantitative assessment of the risk of YFV transmission in Lazio, the central Italian region where the metropolitan city of Rome is located and where the largest arboviral outbreak in continental Europe occurred in summer 2017 [8]. To do so, we adapted a stochastic transmission model, previously developed to assess the transmission risk of chikungunya virus (CHIKV) [9] in the same area, to account for relevant epidemiological dynamics of YFV, using existing field data on *A*. *albopictus* abundance [10] and biting rate on humans [11].

## Study sites

The assessment of YFV transmission risk was carried out on 18 sampling sites placed along a 70 km transect representing a wide range of ecological landscapes, from low–human-population density areas (coastal and rural sites) to highly urbanized areas (metropolitan city of Rome). The site-specific vector abundances over time were characterized by calibrating a mosquito population model against observed mosquito captures [8], taking as input the average daily temperature [12]. The average vector density between July and September was estimated to range between 154 and 4,866 female mosquitoes/ha, whereas the human density within sampling sites ranged from 5 to 267 inhabitants/ha. The selected sites covered a wide range of the vector-to-host ratio (4 to 138 female mosquitoes per inhabitant; see S1 Fig).

## Transmission dynamics

The stochastic transmission model previously developed for CHIKV [9] was adapted to model YFV transmission. Briefly, the overall model includes a temperature-driven model providing the abundance of *A*. *albopictus* and was calibrated to mosquito-capture data, coupled with a disease-transmission model, and was informed with available estimates on epidemiological parameters for YFV and was initialized with a single imported infection in a fully susceptible population. At the end of a patient’s incubation period, we sampled the occurrence of clinical symptoms from a binomial distribution. In baseline simulations, we assumed that only symptomatic patients (including both mild and severe cases) are able to transmit the virus. Fatal outcome was modeled as a binomial process at the end of the infectious period for symptomatic individuals, given that severe symptoms develop in a small fraction of patients only after the end of the viremic period [13]. The outcome of the importation of a single infected case was evaluated at different times between May 1st and November 15th. To account for both stochastic effects and the uncertainty in epidemiological parameters, we evaluated model simulations by repeatedly sampling parameters from known distributions. The total number of simulations for each study site was set to 30,000 (i.e., about 1,000 per week of importation). Full details on the model’s structure are described in the S1 File.

We estimated the basic reproduction numbers over time, the probability of occurrence of an outbreak, and the expected number of yellow fever (YF) cases and fatalities, under the assumption of no disease control (either by vaccination or by vector management interventions).

The basic reproduction number R_0_ represents the average number of secondary cases transmitted by a typical infector during the entire period of infectivity; a value larger than 1 implies potential for a sustained epidemic in the population. R_0_ was computed for different times of importation from standard equations [14,15], after adjusting to consider only symptomatic cases (see S2 File). R_0_ never exceeded the epidemic threshold in urban sites. However, some coastal and rural sites had an average value of R_0_ of about 2.1 at the peak, with an epidemic season (i.e., the time span over which R_0_ exceeds 1) that extended from mid-July to the end of September.

**Fig 1:**
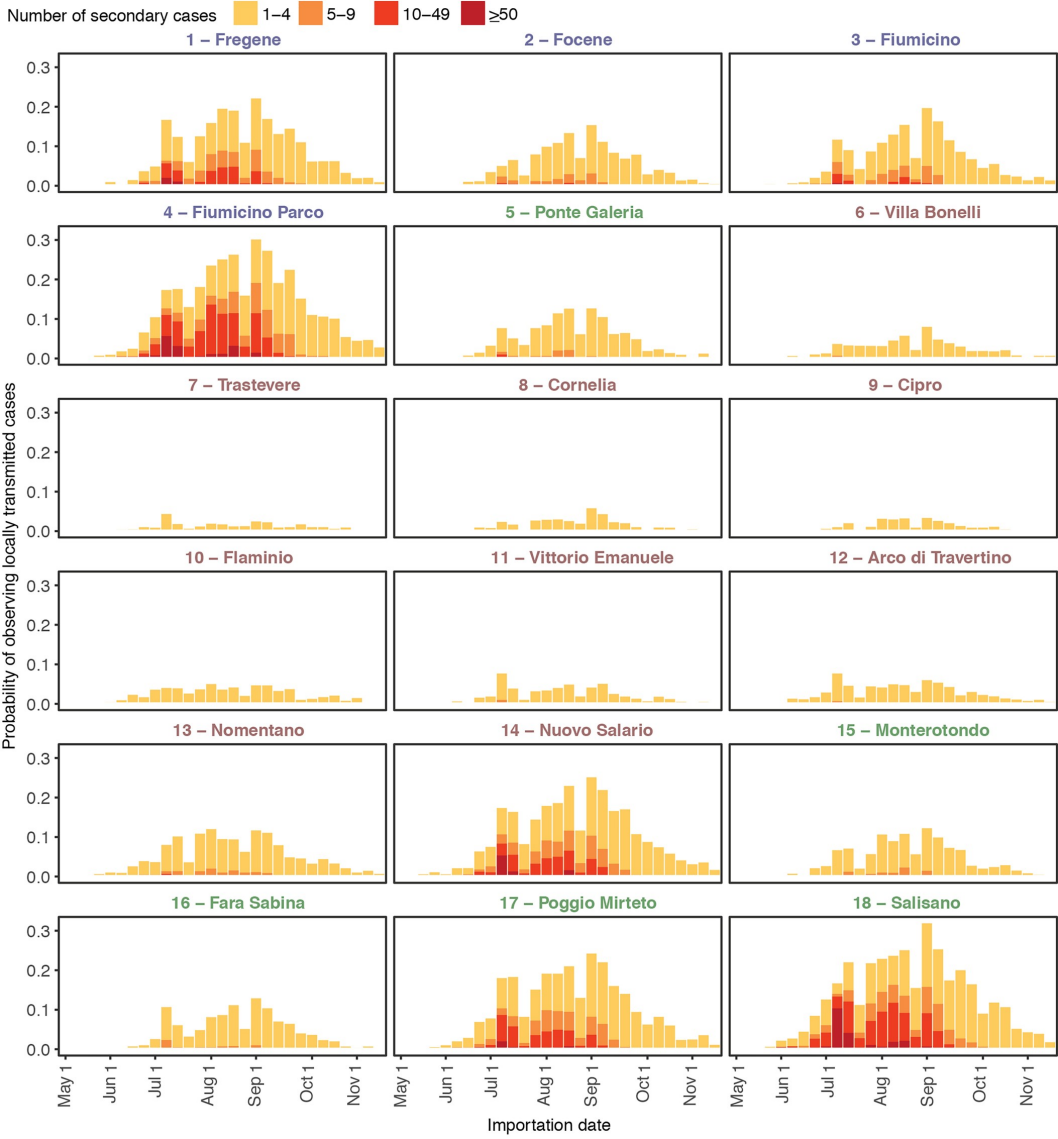
Autochthonous symptomatic YF cases. Probability of autochthonous symptomatic YF cases estimated by the model in 18 sites in Lazio region (Italy), conditional to the introduction of a single imported case at different times of the year and disaggregated by number of secondary cases. Font colors for site IDs represent the geographic classification of the site. Blue: coastal, red: urban, green: rural. YF, yellow fever.

Depending on the site, the average probability of autochthonous symptomatic YF cases throughout the study period ranged between 1.0% and 11.8% (Fig 1); large outbreaks, here defined as those involving more than 50 symptomatic cases, were unlikely (less than 0.65% in all sites). The risk of YFV autochthonous transmission was uneven during the mosquito breeding season but remained relatively stable between mid-July and mid-September in all sites. However, the probability of occurrence of a large outbreak was higher for importations occurring in mid-July, due to the broader time window during which transmission was possible. Coastal and rural sites, characterized by high vector-to-host ratios, had a higher probability of autochthonous transmission (up to 33.1%) and large outbreaks (up to 9.0%) compared to metropolitan sites (maximum probability of autochthonous transmission: 25.9%; maximum probability of large outbreaks: 4.0%). The average probability of observing at least one death ranged between 7.1% and 12.3% and that of observing more than 10 deaths was below 0.5% for all sites; however, peak probabilities of observing at least one death reached 27.6% for importations occurring in mid-July in coastal and rural sites (see S2 Fig).

## Discussion

Given the observed importation of YFV in Europe via infected travelers [5] and the laboratory competence of European *A*. *albopictus* populations to transmit this virus [6], we quantified the risk of YFV transmission for Lazio, Italy, a region that was affected by a CHIKV outbreak during the summer of 2017 [9]. We showed that, given one imported case in urban areas, the risk of transmission is generally low and limited to sporadic cases. However, for some coastal and rural sites there is a nonnegligible potential for large outbreaks, especially if importation occurs during the second half of July. In practice, given the severity of disease caused by YFV, it is likely that ongoing local transmission will be promptly identified and limited by integrated control measures (not included in our model); nonetheless, this result confirms the importance of early outbreak detection capacity [16]. As previously shown for CHIKV [9], the higher risk in coastal and rural sites is related to lower human population densities, which tend to increase the vector-to-host ratio. One of the rural sites with highest estimated YFV transmission risk is Fiumicino, where the largest Italian international airport is located. This increases the chance of presence of potentially infected travelers compared to other rural areas. It is relevant to note that our estimates of vector abundance and vector mortality rates are based on temperatures recorded during 2017. In addition, a precise quantification of risks is subject to many uncertainties on epidemiological parameters of YFV transmission in European *A*. *albopictus* mosquitoes. These uncertainties include, but are not limited to, their dependence on temperature, potential variability across different strains of the virus, and deviations between laboratory measures and actual conditions in the field.

Another open question concerns the potential infectiousness of asymptomatic cases, which is difficult to prove as they are mostly identified a posteriori using serological investigations. Relaxing the assumption that asymptomatic individuals do not transmit and assuming in the extreme opposite case that they transmit at the same rate as symptomatic patients, the estimated risks would be much higher. For example, the peak probability of local transmission would exceed 60% in some metropolitan sites and 80% in coastal and rural ones.

Recent estimates suggest that two YF cases were imported in Italy in 2017 [3]; the number of imported cases for 2018 might be slightly larger due to a higher incidence of infection in Brazil in 2018 (by about 50%) [2]. Imported cases are more likely to arrive during the Brazilian summer, which corresponds to the European winter, when mosquito populations are not active, and in crowded urban areas with lower vector-to-host ratios. This largely reduces the likelihood of an importation in Italy at a time and site of favorable conditions for transmission. Nonetheless, YF cases were confirmed in Brazil throughout June through September 2017, so the possibility of importation during the European summer is not to be completely discarded. Furthermore, many other areas of the world are at risk of YFV outbreaks because of their international connectivity and insufficient vaccination coverage [4]: the possible expansion of YFV to countries with year-round (rather than seasonal) transmission might significantly increase the chances of importation in Europe during the summer in the near future.

Finally, we note that the estimated risk of locally transmitted symptomatic cases of YFV in the considered study area is in the same order of magnitude of dengue (see S3 File). Although introductions of dengue virus (DENV) are currently much more frequent (and indeed local transmission of DENV has repeatedly been detected in France and Croatia [17]), the higher severity of YF and the intrinsic stochasticity by which cases arrive over time and space suggest that the risk of local YFV transmission should not be neglected.

## Conclusion

Overall, the present work reveals a low, but nonnegligible risk of YFV transmission in European areas characterized by substantial *A*. *albopictus* infestation and medium-to-low human density. Considering the severity of YF, this result highlights the need for public health authorities to ensure early diagnosis (not trivial since YF has not been reported in Italy since the 19th century [18]), prompt notification of infected cases and swift responses targeting mosquito populations through vector control interventions and the human population via reactive vaccination campaigns. The recent unexpected rise of YF has caused a worldwide shortage in vaccine stockpiles, which has led to the adoption of fractional dosing immunization during the recent Brazilian outbreaks [19]. Although this approach seems to have been effective in controlling the epidemics locally, many questions remain open [19]. More generally, the availability of YFV vaccine stockpiles at the global scale may be an important challenge to outbreak control in the future. For these reasons, public health authorities might consider preventive general-purpose risk reduction measures such as larviciding, given their demonstrated cost-effectiveness in simultaneously preventing outbreaks of different arboviruses, even in areas with limited risks [20].

## Supporting information

S1 Fig Map of the study sites. Location of the 18 sites for which mosquito abundance estimates were available. The study sites are located along a 70 km transect encompassing the metropolitan city of Rome, Lazio region, Italy. Four sticky traps were placed within each site and weekly mosquito collection lasted from July to November 2012 [9]. The area of circles represents the estimated peak vector-to-host ratio of each site, averaged across the period July through September. Dark grey areas indicate human density higher than 10 inhabitants/ha. Base layers elaborated from ISTAT data (https://www.istat.it). Spatial data processing and map layout generation were done using QGIS (https://www.qgis.org). ISTAT, Istituto Nazionbale di Statistica; QGIS, Quantum Geographic Information System.
(TIF)

S2 Fig YF fatal cases. Probability of fatal outcome due to autochthonous YF transmission estimated by the model in 18 sites in Lazio region (Italy), conditional to the introduction of a single imported case at different times of the year and disaggregated by the number of expected deaths. YF, yellow fever.
(TIFF)

S1 File YF model description. YF, yellow fever.
(PDF)

S2 File YF basic reproductive number (R_0_). YF, yellow fever.
(PDF)

S3 File Results for dengue transmission. (PDF)
