# Supplementary figures and images for: Assessing the risk of autochthonous yellow fever transmission in Lazio, central Italy
Source: PLoS Negl Trop Dis. 2019 Jan 10;13(1):e0006970. doi: 10.1371/journal.pntd.0006970 (PMC6328239; doi:10.1371/journal.pntd.0006970)

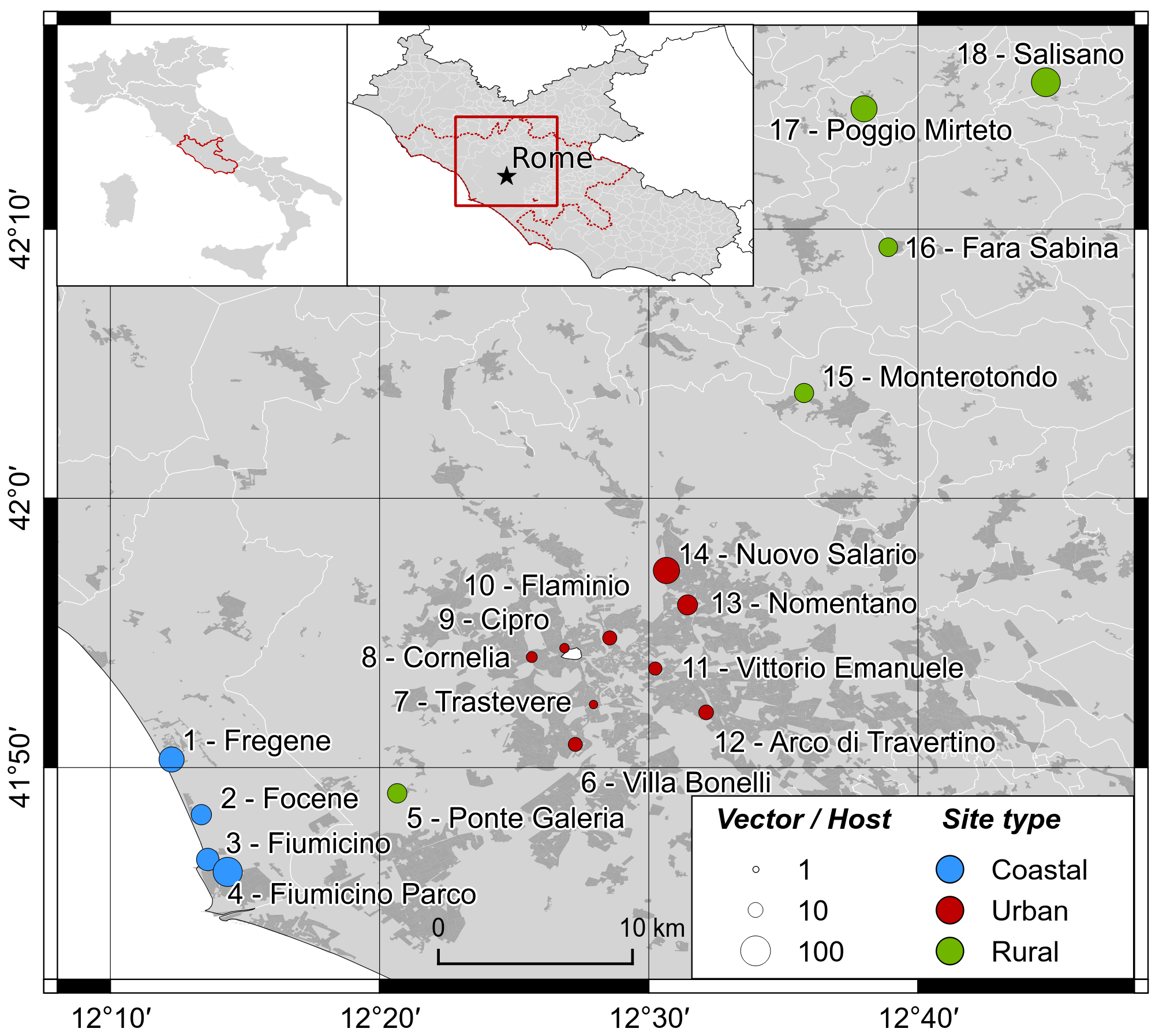

Supplement: S1 Fig — Location of the 18 sites for which mosquito abundance estimates were available. The study sites are located along a 70 km transect encompassing the metropolitan city of Rome, Lazio region, Italy. Four sticky traps were placed within each site and weekly mosquito collection lasted from July to November 2012 [9]. The area of circles represents the estimated peak vector-to-host ratio of each site, averaged across the period July through September. Dark grey areas indicate human density higher than 10 inhabitants/ha. Base layers elaborated from ISTAT data (https://www.istat.it). Spatial data processing and map layout generation were done using QGIS (https://www.qgis.org). ISTAT, Istituto Nazionbale di Statistica; QGIS, Quantum Geographic Information System. (TIF) [file pntd.0006970.s001.tif]

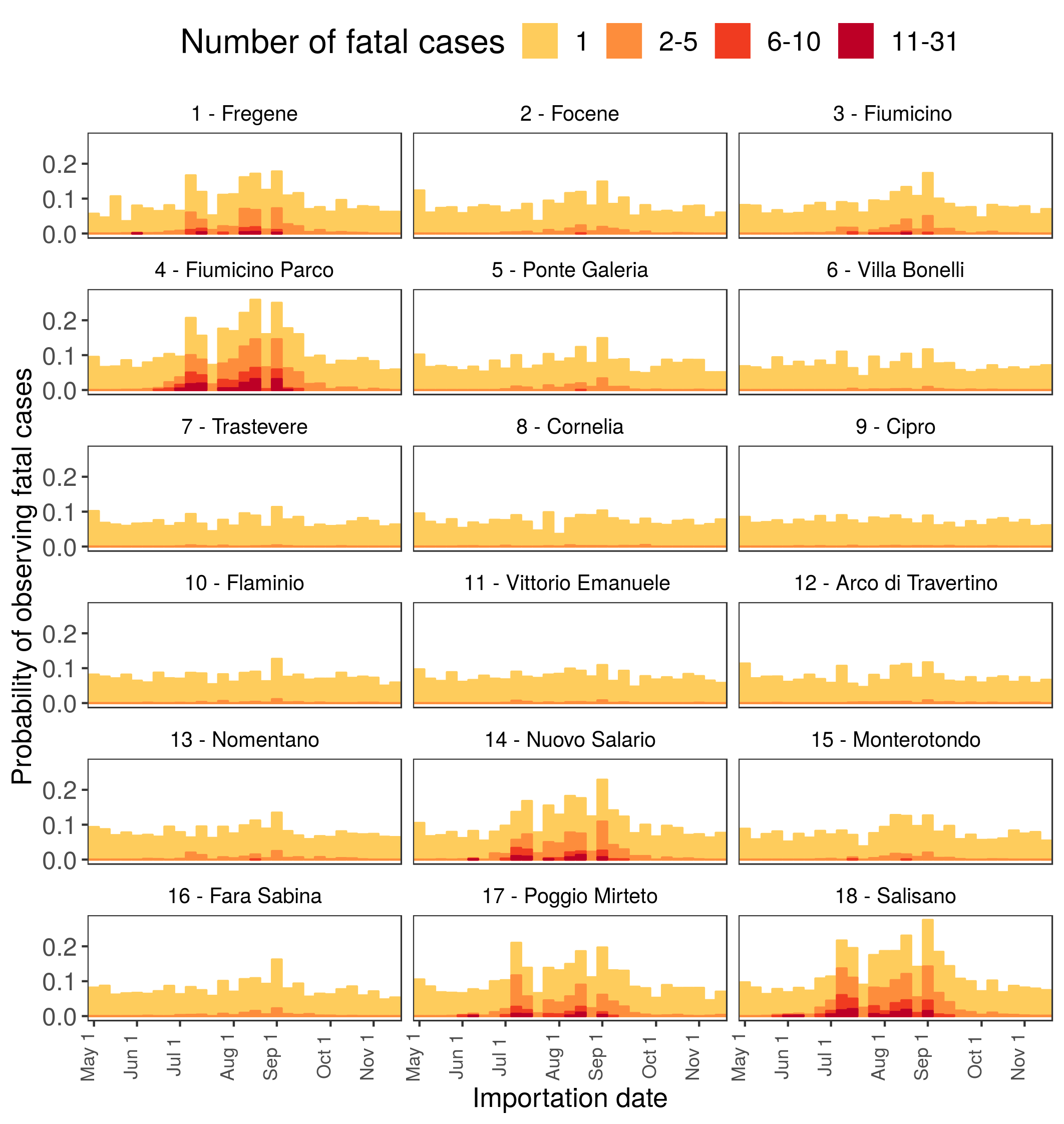

Supplement: S2 Fig — Probability of fatal outcome due to autochthonous YF transmission estimated by the model in 18 sites in Lazio region (Italy), conditional to the introduction of a single imported case at different times of the year and disaggregated by the number of expected deaths. YF, yellow fever. (TIFF) [file pntd.0006970.s002.tiff]
