# Supplementary material for: Assessing the risk of autochthonous yellow fever transmission in Lazio, central Italy
Source: PLoS Negl Trop Dis. 2019 Jan 10;13(1):e0006970. doi: 10.1371/journal.pntd.0006970 (PMC6328239; doi:10.1371/journal.pntd.0006970)
Supplement: S1 File — YF, yellow fever. (PDF) [file pntd.0006970.s003.pdf]

YF model framework

Our model framework includes a temperature-driven model, providing the abundance of *Ae. albopictus* and that was calibrated using mosquito capture data, coupled with a disease transmission model, that was informed with available estimates on epidemiological parameters for YF and initialized with a single imported infection in a fully susceptible population (Fig. S2).

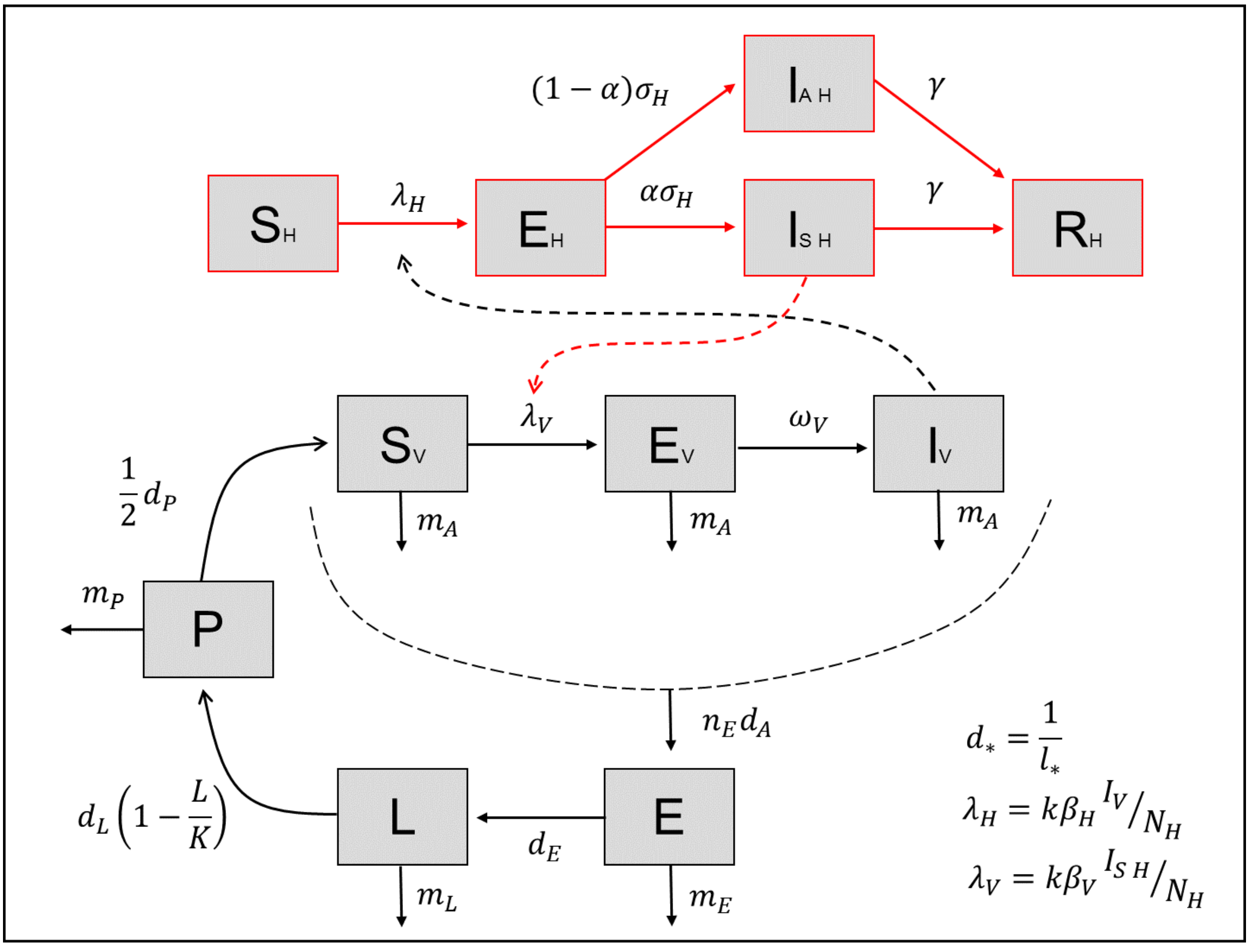

Figure S2. Model flow chart. Model schema describing the evolution of the four life stages of the vector (eggs, E, larvae, L, pupae, P, adult females S_V, E_V, I_V, $N_V = S_V + E_V + I_V$), coupled to the epidemic transmission model (in vectors: susceptible, S_V, latent, E_V, infectious, I_V and in hosts susceptible, S_H, latent, E_H, symptomatic infectious, I_{SH}, asymptomatic infectious, I_{AH}, recovered R_H, $N_H = S_H + E_H + I_{SH} + I_{AH} + R_H$).

The model used in this work is a stochastic implementation of the following system of Ordinary Differential Equations:

$$\begin{cases}\dot{E} = d_A n_E(S_V + E_V + I_V) - (m_E + d_E)E \\ \dot{L} = d_E E - \left(m_L\left(1 + \frac{L}{K}\right) + d_L\right)L \\ \dot{P} = d_L L - (m_P + d_P)P \\ \dot{S}_V = \frac{1}{2} l_P P - (m_A + \lambda_V)S_V \\ \dot{E}_V = \lambda_V S_V - (m_A + \omega_V)E_V \\ \dot{I}_V = -m_A I_V + \omega_V E_V \\ \dot{S}_H = -\lambda_H S_H \\ \dot{E}_H = \lambda_H S_H - \sigma_H E_H \\ \dot{I}_{SH} = \alpha\sigma_H E_H - \gamma I_{SH} \\ \dot{I}_{AH} = (1-\alpha)\sigma_H E_H - \gamma I_{AH} \\ \dot{R}_H = \gamma(I_{SH} + I_{AH})\end{cases}$$

Where: E = mosquito eggs, L = larvae, P = pupae, S_V = adult susceptible vector females, E_V= adult latent vector females, I_V = adult infected vector females, S_H = susceptible hosts, E_H = latent hosts, I_{SH} = symptomatic infectious hosts, I_{AH} = asymptomatic infectious hosts, R_H= recovered hosts.

YF model parametrization

Parameters for the vector abundance model

Mortality (μ_*) and development (l_*) rates for the mosquito life-cycle were assumed to depend on the daily mean temperature T [9], according to equations previously developed [14] from experimental data [21]. The following equations reports the average lengths for different mosquito development stages (*E* = eggs, *L* = larvae, *P* = pupae, *A* = adults):

$l_E(T) = 6.9 - 4e^{-\left(\left(\frac{T-20}{4.1}\right)^2\right)}$,

$l_L(T) = 0.12T^2 - 6.6T + 98$,

$l_P(T) = 0.027T^2 - 1.7T + 27.7$,

$l_A(T) = 0.046T^2 - 2.77T + 45.3$.

Mosquito development rates were computed as $d_* = 1/l_*$

Equations for stage-specific mosquito mortality rates (*E* = eggs, *L* = larvae, *P* = pupae, *A* = adults) were the following:

$m_E(T) = 506 - 506e^{-\left(\left(\frac{T-25}{27.3}\right)^6\right)}$,

$m_L(T) = 0.029 + 858e^{T-43.4}$,

$m_P(T) = 0.021 + 37e^{T-36.8}$,

$m_A(T) = \theta(0.031 + 95820e^{T-50.4})$.

The θ parameter represents a decrease in mosquito longevity under field conditions with respect to laboratory conditions; K represents a carrying capacity on the larvae stage. Both parameters were estimated by fitting seasonal mosquito capture data from 2012 in the 18 study sites and site-specific temperatures from the same year.

Parameters for YF transmission

The transmission model was parametrized using the most recent estimates on yellow fever transmission parameters available for European strain of *Aedes albopictus.* All relevant parameters are listed in Table S1.

Table S1 Epidemiological parameters used for the YF transmission model.

**Parameter**	**Unit**	**Distribution**	**Parameters**	**Reference**
Date of imported infection	Date	Uniform	1 May; 15 Nov	-
Mosquito biting rate (k)	Bites/ mosquito/ day	Uniform	Min: 0.08; Max: 0.10	[9,11]
Probability of vector-to-human transmission per bite (β_H)	%	Uniform	Min: 0; Max: 22	[6]
Probability of human-to-vector transmission per bite (β_V)	%	Uniform	Min: 33; Max:75	[6]
Extrinsic incubation period ($1/\omega_V$)	Days	Uniform	Min: 8; Max: 14	[6, 22]
Intrinsic incubation period ($1/\sigma_H$)	Days	Lognormal	Mean = 4.6; Var = 2.7	[3, 23]
Human infectious period ($1/\gamma$)	Days	Normal	Mean = 4.5; Var = 0.6	[3, 24]
Probability of developing symptoms (α)	%	Uniform	Min: 26; Max: 63	[25]
Probability of fatal outcome for symptomatic cases (μ)	%	Uniform	Min: 5; Max: 28	[3]

The choice for the extrinsic incubation period (EIP) range was based on two experimental studies [6, 22]. As in both experiments no mosquitoes were found infected before 7 days while Miller et al. (who used an American strain of *Ae. albopictus*) suggested an EIP of 9 days, we conservatively selected as a lower bound for the EIP a value of 8 days. For what concerns the upper bound, Amraoui et al. [6] found similar proportions of infected mosquitoes at 14 and 21 days, suggesting that 14 days may be sufficient for all mosquitoes to develop infection.

References

21. Delatte H, Gimonneau G, Triboire A, Fontenille D. Influence of temperature on immature development, survival, longevity, fecundity, and gonotrophic cycles of *Aedes albopictus*, vector of chikungunya and dengue in the Indian Ocean. J Med Entomol. 2009; 46: 33–41
22. Miller BR, Mitchell CJ, Ballinger ME. Replication, tissue tropisms and transmission of yellow fever virus in *Aedes albopictus*. Trans R Soc Trop Med Hyg. 1989; 83 (2), 252-255. doi: 10.1016/0035-9203(89)90667-6
23. Johansson MA, Arana-Vizcarrondo N, Biggerstaff BJ, Staples JE. Incubation periods of Yellow fever virus. Am J Trop Med Hyg. 2010; 83(1):183-8. DOI: 10.4269/ajtmh.2010.09-0782
24. Monath TP. Yellow fever: an update. Lancet Infect Dis. 2001; 1(1):11-20. doi: 10.1016/S1473-3099(01)00016-0
25. Johansson MA, Vasconcelos PF, Staples JE. The whole iceberg: estimating the incidence of yellow fever virus infection from the number of severe cases. Trans R Soc Trop Med Hyg 2014; 108(8):482-7. doi: 10.1093/trstmh/tru092
