# Supplementary material for: Assessing the risk of autochthonous yellow fever transmission in Lazio, central Italy
Source: PLoS Negl Trop Dis. 2019 Jan 10;13(1):e0006970. doi: 10.1371/journal.pntd.0006970 (PMC6328239; doi:10.1371/journal.pntd.0006970)
Supplement: S2 File — YF, yellow fever. (PDF) [file pntd.0006970.s004.pdf]

YF basic reproductive number (R_0)

Fig. S3 shows the variation of the basic reproductive numbers throughout the mosquito breeding season in the 18 study sites. Average and confidence interval values were computed, using observed temperatures from 2017, from 30000 stochastic runs of the mosquito population model, each with a different sample from estimated posterior distributions of the parameters K and θ and from prior distributions of epidemiological parameters.

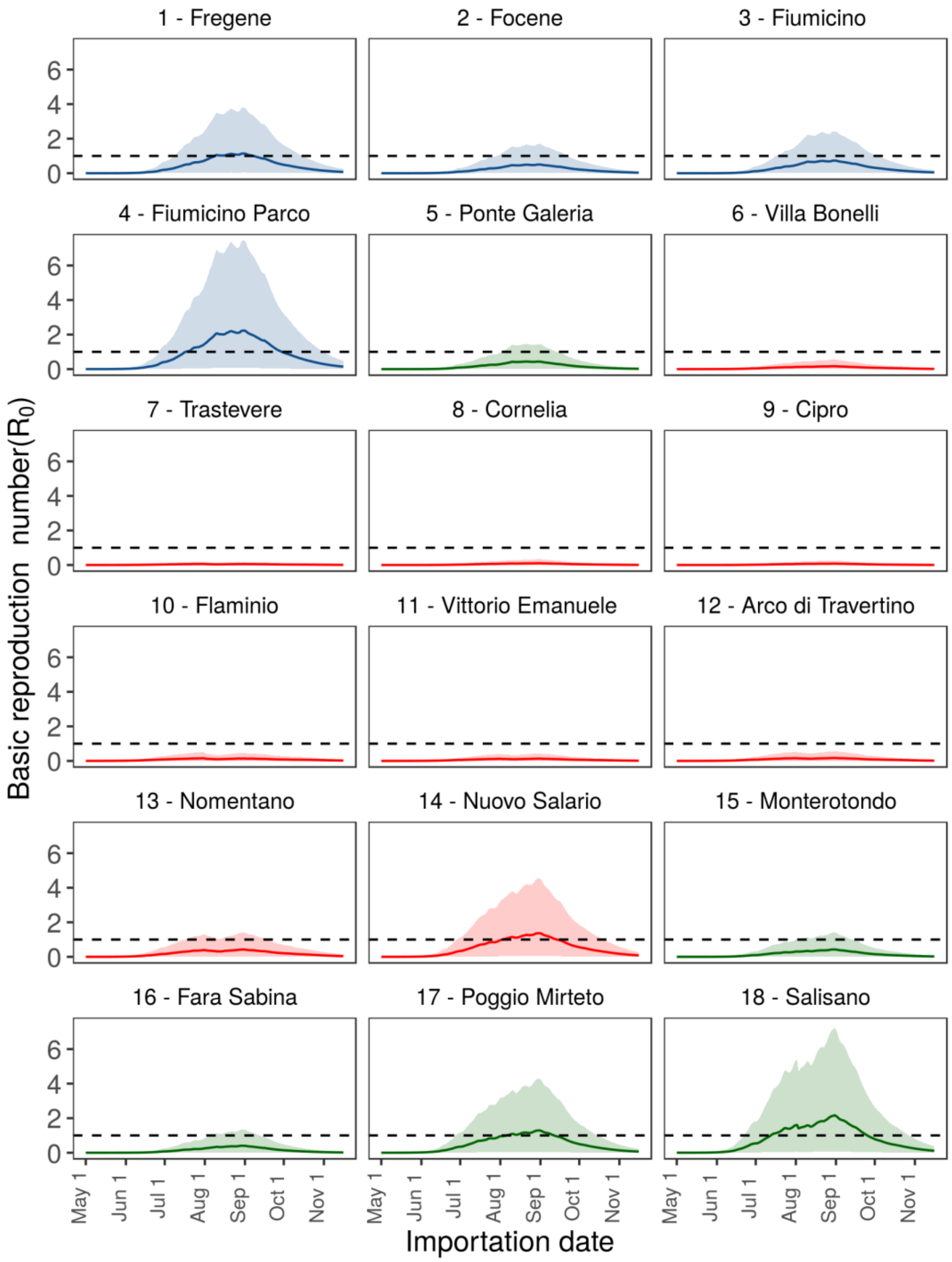

Figure S3. YF basic reproductive number, R_0.

Basic reproductive number $R_0(t)$ for each site, adjusted to consider only YF symptomatic cases. Solid lines represent average values across simulations. Shaded areas represent the 95% confidence intervals. Colours represent the geographic classification of the site. Blue: coastal, red: urban, green: rural (see S1 Fig).

The basic reproductive number for YF was computed as follows:

$$R_0(t) = R_0^{HV}(t)\, R_0^{VH}(t) = \left(\alpha k \beta_V \frac{N_V(t)\omega_V}{\gamma N_H\big(\omega_V + m_A(T(t))\big)}\right)\left(\frac{\alpha k \beta_H}{m_A(T(t))}\right)$$

where $N_v(t)$ is the vector abundance over time ($N_V = S_V + E_V + I_V$), N_H is the human population density ($N_H = S_H + E_H + I_{SH} + I_{AH} + R_H$) and m_A is the mosquito mortality, dependent on the time-varying temperature T(t) [14]; see Table S1 in S2 Text for the meaning and values of other epidemiological parameters.
