# Supplementary material for: Assessing the risk of autochthonous yellow fever transmission in Lazio, central Italy
Source: PLoS Negl Trop Dis. 2019 Jan 10;13(1):e0006970. doi: 10.1371/journal.pntd.0006970 (PMC6328239; doi:10.1371/journal.pntd.0006970)
Supplement: S3 File — (PDF) [file pntd.0006970.s005.pdf]

Results for dengue transmission

The same model framework was also tailored to dengue transmission by considering appropriate distributions for all relevant dengue epidemiological parameter, see Table S2 for further details.

Table S2 Epidemiological parameters used for the dengue transmission model.

Parameter	Unit	Distribution	Parameters	Reference
Probability of vector-to-human transmission per bite (β_H)	%	Uniform	Min: 0; Max: 61.5	[26,27]
Probability of human-to-vector transmission per bite (β_V)	%	Uniform	Min: 9.3; Max: 44.8	[26,27]
Extrinsic incubation period ($1/\omega_V$)	Days	Uniform	Min: 8; Max: 14	[26,27]
Intrinsic incubation period ($1/\sigma_H$)	Days	Constant	2 days	[28]
Human infectious period ($1/\gamma$)	Days	Constant	4 days	[28]
Probability of developing symptoms (α)	%	Truncated Normal	Mean = 0.23; Var = 0.002;	[29]
Probability of fatal outcome for symptomatic cases (μ)	%	Beta	α = 6.66, β = 505.58	[30]

Result showed that the probability of observe autochthonous symptomatic cases of dengue is lower compared to yellow fever, as shown by Fig. S5. Depending on the site, this probability averaged over the study period ranged between 0.24 and 4.51%. Sites characterized by high vector-to-host ratios had a higher weekly probability of occurrence of autochthonous transmission, up to 12.9% in coastal and rural sites and 9.8% in metropolitan sites. Outbreaks involving more than 50 cases were highly unlikely. The risk of dengue autochthonous transmission peaked late during the mosquito breeding season, in early September. However, the probability of outbreaks involving more cases was higher for importations occurring earlier, from mid-June rather than September, due to the broader time window during which transmission could be viable. The estimated dengue basic reproductive number for symptomatic transmission was lower than yellow fever (Fig. S6), being consistently under the epidemic threshold in urban sites. Finally, the average probability of observing at least one death ranged between 0.26 and 0.54% and no more than three fatal outcomes in a site have been observed during the simulations. However, peak probabilities of observing at least one death reached 1.47% for importations occurring between mid-July and early August (Fig. S7).

The estimated risks would be much higher if asymptomatic individuals transmit at the same rate as symptomatic patients. For example, the peak probability of local transmission would reach about 40% and 60% in some metropolitan and coastal/rural sites, respectively, while the average probability of observing

autochthonous symptomatic cases over the study period would increase to 24.2%. This high probability does not seem to be compatible with the high number of dengue importations in the region (about 10-20/year) and the absence of detection of locally transmitted cases [31].

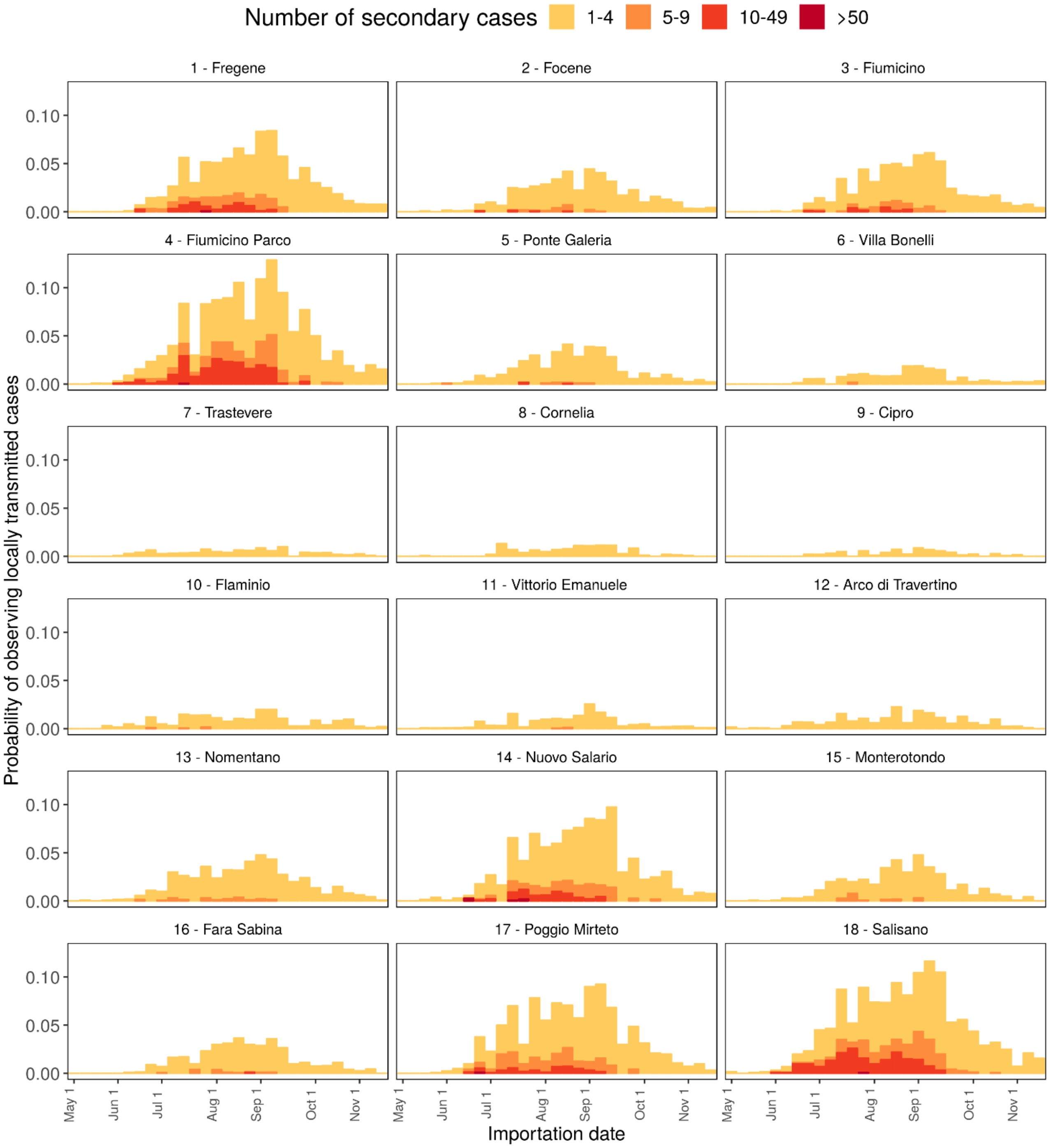

Figure S5. Autochthonous symptomatic dengue cases. Probability of autochthonous symptomatic dengue cases estimated by the model in 18 sites in Lazio region (Italy), conditional to the introduction of a single imported case at different times of the year and disaggregated by number of secondary cases.

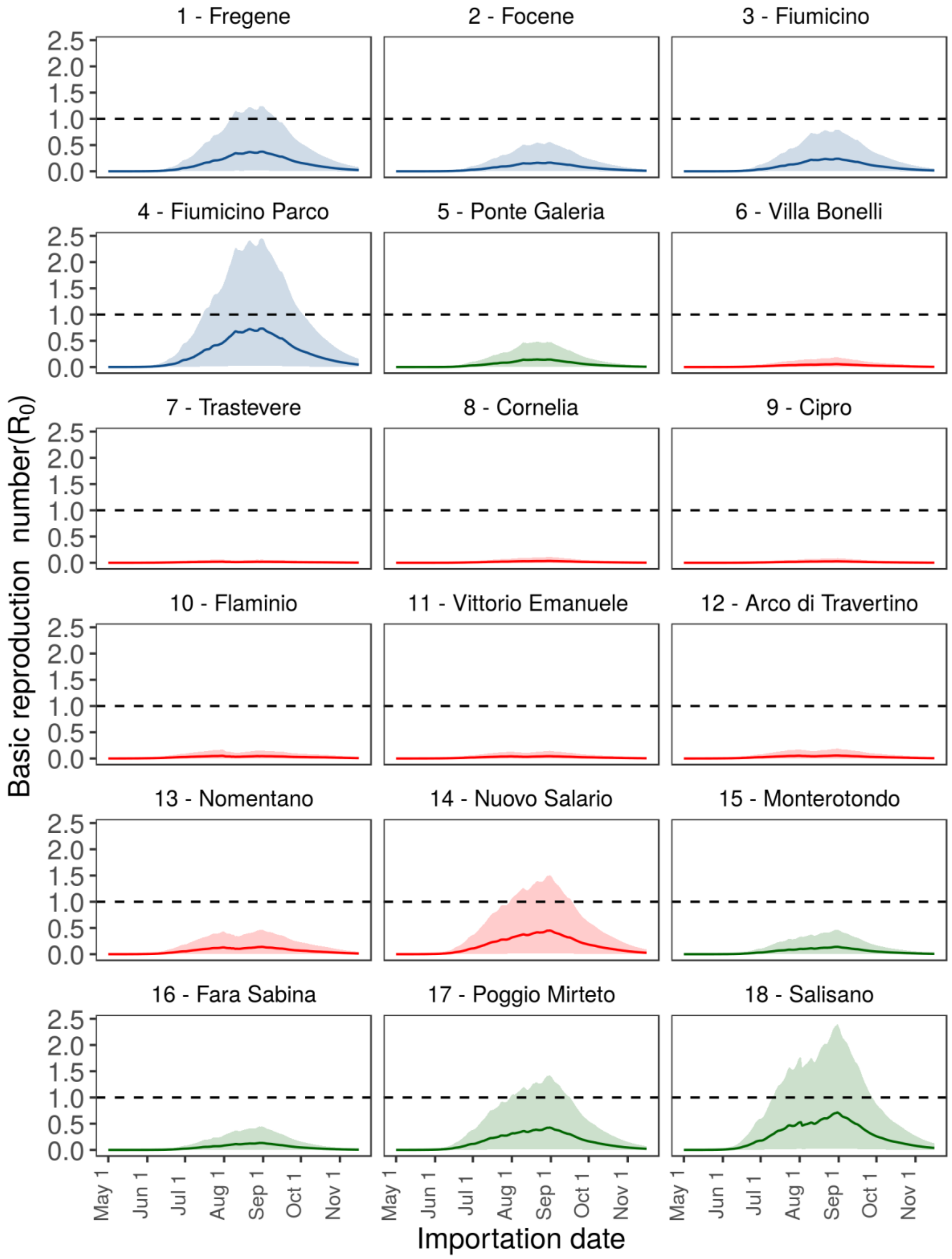

Figure S6. Dengue basic reproductive number, R_0. Basic reproductive number $R_0(t)$ for each site, adjusted to consider only dengue symptomatic cases. Solid lines represent average values across simulations. Shaded areas represent the 95% confidence intervals. Colours represent the geographic classification of the site. Blue: coastal, red: urban, green: rural.

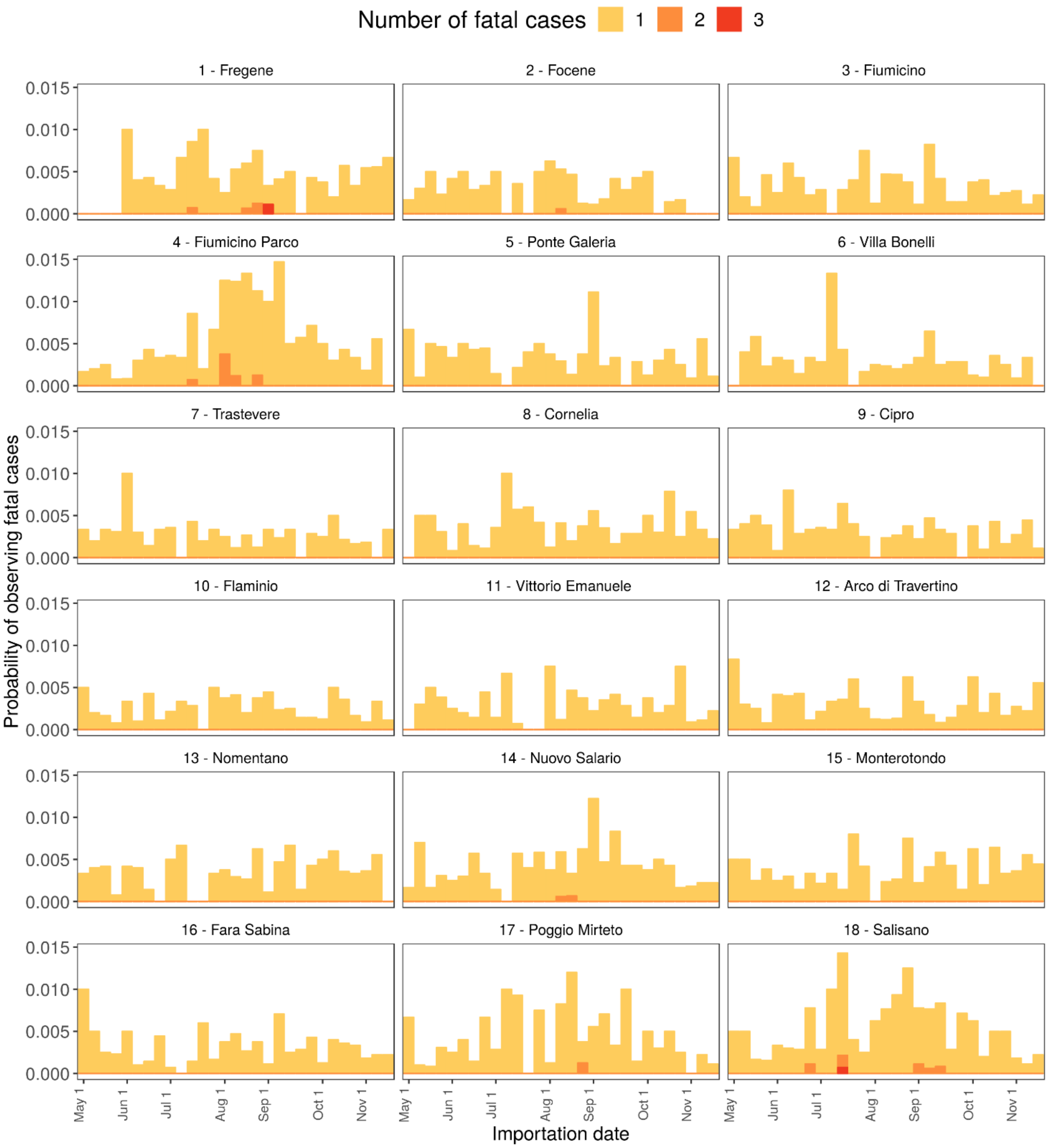

Figure S7. Dengue fatal cases. Probability of fatal outcome due to autochthonous dengue transmission estimated by the model in 18 sites in Lazio region (Italy), conditional to the introduction of a single imported case at different times of the year and disaggregated by the number of expected deaths.

References

26. Vega-Rua A, Zouache K, Caro V, Diancourt L, Delaunay P, Grandadam M, Failloux AB. High efficiency of Temperate *Aedes albopictus* to transmit chikungunya and dengue viruses in the southeast of France. PLoS ONE 2013; 8(3): e59716. doi: 10.1371/journal.pone.0059716
27. Brustolin M, Santamaria C, Napp S, Verdún M, Rivas R, Pujol N, Talavera S, Busquets N. Experimental study of the susceptibility of a European *Aedes albopictus* strain to dengue virus under a simulated Mediterranean temperature regime. Med Vet Entomol. 2018; doi:10.1111/mve.12325
28. Lourenço J, Recker M. The 2012 Madeira dengue outbreak: epidemiological determinants and future epidemic potential. PLoS Negl Trop Dis. 2014; 8(8):e3083.
29. Grange L, Simon-Loriere E, Sakuntabhai A, Gresh L, Paul R, Harris E. Epidemiological risk factors associated with high global frequency of inapparent dengue virus infections. Front Immunol. 2014; 5(280). doi: 10.3389/fimmu.2014.00280.
30. Global Burden of Disease 2004 update. Geneva.
31. Ministero della Salute. Piano Nazionale di sorveglianza e risposta alle arbovirosi trasmesse da zanzare invasive (Aedes sp.) con particolare riferimento ai virus Chikungunya, Dengue e Zika - 2018.
